# ASSOCIATION OF ASSISTED LIVING HOMES PREPAREDNESS FOR AND RESPONSES TO THE COVID-19 PANDEMIC WITH RESIDENT PAIN

**DOI:** 10.1093/geroni/igad104.1630

**Published:** 2023-12-21

**Authors:** Shovana Shrestha, Colleen Maxwell, Hana Dampf, Rashmi Devkota, Matthias Hoben

**Affiliations:** University of Alberta, Edmonton, Alberta, Canada; University of Waterloo, Waterloo, Ontario, Canada; University of Alberta, Edmonton, Alberta, Canada; University of Alberta, Edmonton, Alberta, Canada; York University, Toronto, Ontario, Canada

## Abstract

The COVID-19 pandemic severely disrupted care processes in assisted living (AL) and negatively affected resident outcomes associated with resident pain (e.g., mobility problems, depression). Pain has severe consequences, including hopelessness, insomnia, depression, poor quality of life. However, we lack research on how the pandemic affected AL resident pain. To address this gap, we linked surveys from 42 AL homes in Alberta, reflecting pandemic waves 1 (Mar-Jul 2020) and 2 (Nov 2020-Feb 2021), to the Resident Assessment Instrument (RAI) records of 1,828 residents (wave 1: 890, wave 2: 938) who lived in these homes during these periods. Using generalized estimating equation models, we assessed whether resident characteristics, physical and occupational therapy received, home preparedness for and responses to the pandemic were associated with resident pain (measured as at least moderate daily pain or pain of excruciating intensity, based on the RAI pain items). Over 19% of the residents reported pain (wave 1: 19%, wave 2: 19.1%). Resident characteristics associated with pain were cognitive impairment (OR=0.4, 95% CI: 0.3-0.6), loneliness (OR=1.8, 95% CI: 1.3-2.6), arthritis (OR=2.1, 95% CI: 1.6-2.8), fractures (OR=1.97, 95% CI: 1.4-2.9), polypharmacy (OR=1.82, 95% CI: 1.3-2.6) and use of analgesics (OR=1.76, 95% CI: 1.3-2.3). Home preparedness and physical and occupational therapy received were not associated with pain, but more communication with family/friend caregivers (OR=0.41, 95% CI: 0.2-0.8) was. Effective communication of family/friends with residents may promote better management of residents’ pain. Further, longitudinal studies examining resident and AL home characteristics and its impact on residents’ pain are needed.

